# Cardiovascular risk in rural workers and its relation with body mass index

**DOI:** 10.20945/2359-3997000000011

**Published:** 2018-01-01

**Authors:** Joana Carolina Bernhard, Kely Lisandra Dummel, Éboni Reuter, Miriam Beatris Reckziegel, Hildegard Hedwig Pohl

**Affiliations:** 1 Universidade de Santa Cruz do Sul Universidade de Santa Cruz do Sul Departamento de Educação Física e Saúde Santa Cruz do Sul RS Brasil Departamento de Educação Física e Saúde, Universidade de Santa Cruz do Sul (Unisc), Santa Cruz do Sul, RS, Brasil

**Keywords:** Agribusiness, mortality, cardiovascular diseases

## Abstract

**Objective:**

Evaluate the propensity of cardiovascular risk in rural workers and, through the Framingham Risk Score (FRS), relate this risk with the classification of Body Mass Index (BMI).

**Subjects and methods:**

This study is characterized as descriptive and exploratory, with the participation of 138 subjects, ranging between 25-73 years old. Clinical and laboratory analysis of the risk factors contained in the FRS were performed, in addition to the determination of BMI, blood pressure, smoking and physical inactivity.

**Results:**

The procedures indicated a low risk of a coronary event in 10 years with 70.3% of the population. In contrast, 88.4% of the subjects were overweight. It was evidenced a risk improvement as the BMI increased, since 96.4% of high-risk cases were overweight or obese.

**Conclusion:**

Results suggest larger prevalence of intermediary or high FRS for women with higher BMI, which was not observed in men.

## INTRODUCTION

Cardiovascular diseases are responsible for the high number of deaths, and recorded worldwide data in 2011 of 17 million, since 2000 leading the ranking of the ten leading causes of mortality. Mortality and morbidity levels in adults from 2008 to 2011, reported by WHO, show a significant increase of deaths due to cardiovascular disease, with production losses, and expenses with medical care, treatment and rehabilitation. Predictions are that by 2025 the risk of coronary heart disease and stroke will have increased by 120% for women and 137% for men living in developed countries (1). These figures reinforce the importance of effective primary measures for the prevention of these diseases (2).

Hypertension, diabetes mellitus, dyslipidemia, obesity and some habits related to lifestyle (poor diet, smoking, alcohol consumption, physical inactivity) are factors that predispose to cardiovascular risk (3). The Brazilian Society of Cardiology data indicate that 80% of individuals of the Brazilian adult population is sedentary and 52% are overweight, 11% obese, which reinforces the propensity of increased morbidity and mortality, since obesity is an independent risk factor associated with cardiovascular diseases (4).

These aspects combined with regional characteristics of the population and the changes processed in the labor world, with numerous effects on health and epidemiological profile of the working population (5), broaden the spectrum of health problems. Because, in addition to unhealthy lifestyles, inadequate working conditions, such as long working hours and high occupational stress, may predispose workers to high risk of cardiovascular diseases (6).

Rural workers have presented health problems. There is evidence that, by underreporting, the prevalence of diseases is higher in rural than in urban areas. Many factors could be related to health worsening, including restricted access to health system and work economic dependence, which subjects workers to long working days, frequent physical efforts, low income, assiduity even at physical adversities, and long term labor (increasing age group for this population) (7). For the International Labour Organization (ILO) rural work is of great significance, and more dangerous than other activities. It is estimated that millions of farmers suffer from serious health problems (8). Therefore, prevention measures are an important issue in promoting the health of these workers.

Given the complexity of these interactions, it is performed, in 1960, the first cohort study focusing on cardiovascular disease by identifying risk factors and pathophysiology, the Framingham Heart Study (FHS), a time when mortality and the incidence of cardiovascular disease showed progressive increases (9). As a result of advances in the FHS, which occurred from 1998, it is possible to stratify the risk of coronary events in 10 years, through a specific score for each risk factor, using clinical variables of daily practice. This indicative stratification of cardiovascular disease risk propensity is classified as low, intermediate and high risk (10). According to the Ministry of Health, the Framingham Score identifies the potential for developing cardiovascular disease even before the onset of symptoms (11).

In this context, the objective of this study is to evaluate the cardiovascular risk in farmers and their relation to the classification of Body Mass Index.

## SUBJECTS AND METHODS

This is a descriptive exploratory study, developed from the database project “Screening of risk factors related to overweight in agribusiness workers using new analytical and health information technologies” developed at the University of Santa Cruz do Sul, approved by the Ethics Committee in Research with Human Beings, under the Protocol 2509/10. We evaluated 138 subjects with a mean age of 51.32 years (SD 10.32), who met the following inclusion criteria: be 18 years old and not be carriers coronary artery disease, stroke, heart failure and acute myocardial infarction.

Labor activities of the sample individuals are those typical of family farming workers, such as planting, farming maintenance, harvesting, animal husbandry, manufacturing of homemade products and trading of the production. Sample was obtained among residents in the municipalities of Santa Cruz do Sul, Passo do Sobrado, Vale Verde, Rio Pardo, Candelária, Encruzilhada do Sul e General Câmara, in the State of Rio Grande do Sul, Brazil. According to Bertê and cols. (12), the region has its rural sector dedicated mainly to monoculture, but receives incentives aiming to diversification of production and development of family agroindustry.

The screening of the subjects was performed by external seminar with the institutions involved in the study. Opportunity in which voluntary participants were informed about the purpose of the study and the conditions of involvement, when the lifestyle questionnaire was applied. Sampling was nonprobabilistic and by convenience.

A survey form proposed by the Brazilian Association of Survey Companies (ABEP – 2014) (13) was used for economic classification, which consists on collecting data of movable and immovable property, domiciliary goods, as well as the graduation level of the householder. There are eight classification levels for Brazilian Criterion: A1, A2, B1, B2, C1, C2, D and E. In this study, subjects were divided in two groups, one of them formed by the members of A1, A2, B1 and B2, and the other one formed by the remaining categories.

Anthropometric, biochemical and physiological assessments were subsequently applied at the University. The body mass index (BMI) was calculated from the body mass obtained on the scale (Welmy) and height measured in a stadiometer, and calculated from dividing weight (kg) by height (m) squared. Systolic blood pressure (SBP) was measured after five minutes rest, with the subject seated, and the data categorized as normal SBP (< 129 mmHg), borderline (between 130 and 139 mmHg) or hypertension (above 140 mmHg) according to the VI Brazilian Guidelines of Hypertension (2010) (14).

Blood tests were performed at the Laboratory of Clinical Biochemistry at the University of Santa Cruz do Sul, using reactive of Labtest brand in semiautomated system (LabMax Progress – LabTest), observing, to the glycemic index the recommendations of the American Diabetes Association (15). Total cholesterol (TC) and high-density lipoprotein cholesterol (HDL-c) reference values of the Brazilian Cardiology Society (16). Blood collection was performed in the brachial vein, preceded by fasting for 12 hours using two *vacutainer*, one of them containing fluoride\oxalate (to obtain plasma) and another without additive (to obtain serum). Blood samples deposited in *vacutainer* without anticoagulants were incubated at 37°C for 15 minutes and centrifuged at 2500 rpm for five minutes to collect serum. The plasma samples were subjected to the determination of glucose (GLU), and the serum samples submitted to the determination of the TC and HDL-c. Diagnosis of diabetes was determined after the results of the glucose test.

According to the criteria proposed by the Framingham Heart Study (FHS) the following risk factors were considered: age, sex, diabetes, TC, HDL-c, smoking and SBP. Through the Framingham score (FRS), the risk of occurrence of coronary events in 10 years was calculated. For each risk factor was applied a specific score as suggested by the FHS, obtaining the final score by the sum of individual points. The results were classified as low risk (LR) score < 10%, intermediate risk (IR) score between 10 and 20% and high risk (HR) > 20%. It is important to highlight that all those identified as diabetics were considered automatically as HR (6).

Data were analyzed using the Statistical Software for Social Sciences (SPSS, version 20.0), using descriptive statistics with central tendency and dispersion measures, for numeric variables, and frequency and percentage, for categorical variables. Pearson's chi-square test was used to the analytical statistics of the frequencies distribution between FRS and BMI as well as between FRS and sociodemographic characteristics, considering p < 0.05. In order to evaluate the prevalence ratio of BMI over the FRS, a Poisson Regression Test was used.

## RESULTS

Most of the subjects is aged 40-55 (44.2%), married (75.4%), from C1-C2-D economic classes (58%), up to 7 years educated (73.2%) and women (62.3%). Regarding BMI, it is noteworthy that 88.4% of employees have some level of overweight (overweight or obese). Obesity prevails among women (51.2%) and overweight among men (65.4%). Regarding the FRS, 81.4% of women and 51.9% of men have low risk (Table 1).

**Table 1:** Characterization of the sample

Variables		Gender		Total* 138 (100)
		Female* 86 (62.3)	Male* 52 (37.7)	
Age range				
	< 40 years	14 (16.3)	11 (21.2)	25 (18.1)
	40-55 years	41 (47.7)	20 (38.5)	61 (44.2)
	> 55 years	31 (36.0)	21 (40.4)	52 (37.7)
Marital status				
	Single	6 (7.0)	8 (15.4)	14 (10.1)
	Married	65 (75.6)	39 (75.0)	104 (75.4)
	Others	15 (17.4)	5 (9.6)	20 (14.4)
Economic level				
	B1-B2	32 (37.2)	26 (50.0)	58 (42.0)
	C1-C2-D	54 (62.8)	26 (50.0)	80 (58.0)
Schooling				
	Up to 7 years	64 (74.4)	37 (71.2)	101 (73.2)
	8 to 10 years	9 (10.5)	6 (11.5)	15 (10.9)
	11 years or more	13 (15.1)	9 (17.3)	22 (15.9)
BMI				
	Recommended range	8 (09.3)	8 (15.4)	16 (11.6)
	Overweight	34 (39.5)	34 (65.4)	68 (49.3)
	Obesity	44 (51.2)	10 (19.2)	54 (39.1)
Total cholesterol				
	Desirable	36 (41.9)	22 (42.3)	58 (42.0)
	Increased	50 (58.1)	30 (57.7)	80 (58.0)
HDL				
	Desirable	75 (87.2)	40 (76.9)	115 (83.3)
	Decreased	11 (12.8)	12 (23.1)	23 (16.7)
SBP				
	Recommended range	34 (39.6)	26 (50.0)	60 (43.5)
	Bordering	22 (25.6)	10 (19.2)	32 (23.2)
	Hypertension	30 (35.0)	16 (30.8)	46 (33.3)
Glucose				
	Desirable	36 (41.9)	20 (38.5)	56 (40.6)
	Pre-diabetes	38 (44.2)	22 (42.3)	60 (43.5)
	Diabetes	12 (14.0)	10 (19.2)	22 (15.9)
FRS				
	Low risk	70 (81.4)	27 (51.9)	97 (70.3)
	Intermediate risk	1 (1.2)	12 (23.1)	13 (9.4)
	High risk	15 (17.4)	13 (25.0)	28 (20.3)

* frequency (percentage); BMI: body mass index; HDL: high density lipoprotein; SBP: systolic blood pressure; FRS: Framingham Risk Score.

Regarding the biochemical characteristics and BP, it can be seen that 58.0% has increased level of TC, HDL values within the desired standard in 83.3% of cases. Yet the glucose levels of 43.5% of workers are in the range of pre-diabetic and 15.9% in the diabetes range (Table 1).

By analyzing the average values of the indicators that compose the estimate of FRS and the average of risk score, we observed that the factors that had higher scores on the FRS were the mean age 51.32 ± 10.32 years and the total cholesterol 211.81 ± 48.98 mg/dL. It is important to state that 93.5% of workers had no smoking habit. As regards the systolic pressure, the mean value obtained was 131.67 ± 17.00 mmHg. The average score of 11.09 ± 6.19 points represents low risk for myocardial infarction or coronary heart disease within 10 years (Table 2) which is confirmed by the percentage of individuals rated as LR (Table 1).

**Table 2:** Average values in the indicators that composse the Framingham Risk Score

FRS variables	Mean value (SD)	Mean score (SD)
Age	51.32 (10.32)	4.75 (5.51)
Cholesterol level	211.81 (48.98)	3.70 (2.55)
Smoke* [yes/no]	9 (6.5)/129 (93.5)	0.35 (1.51)
HDL	49.96 (10.97)	0.48 (0.94)
SBP	131.67 (17.00)	1.83 (1.88)
FRS	11.09 (6.19)	-

* frequency (percentage); FRS: Framingham Risk Score; HDL: high density lipoprotein; SBP: systolic blood pressure; SD: standard deviation.

When stratifying the risk of coronary artery disease, according to the FRS, it was observed that 70.3% of workers has low risk, 8.6% has intermediate risk and 22.3% has high risk When analyzing the risk related to BMI classification, statistical significance was found (p = 0.035) indicating relationship between the variables, while obesity is a preponderant factor in the definition of risk, there are other intervening variables that were not evidenced in this study. Although most of the subjects were rated low risk, it is noteworthy that among subjects with intermediate risk 69.2% are overweight, since those with high risk in 96.4% of the patients present weight excess: overweight (39.3%) or obese (57.1%). Another fact to highlight is the occurrence of high risk in the subject with recommended weight range, which was due to the presence of diabetes mellitus (Table 3).

**Table 3:** Relation between BMI and the Framingham Risk Score

BMI	FRS			p*
	Low risk** 97 (70.3)	Intermediate risk** 13 (9.4)	High risk** 28 (20.3)	
Recommended range	12 (12.4)	3 (23.1)	1 (3.6)	
Overweight	48 (49.5)	9 (69.2)	11 (39.3)	0.035
Obesity	37 (38.1)	1 (7.7)	16 (57.1)	

* Pearson's chi-square;

** frequency (percentage); FRS: Framingham Risk Score; BMI: body mass index.

When analyzing the prevalence ratio between BMI and FRS, it can be observed that overweight women have 14.7% greater risk prevalence than those rated as normal BMI. Obese women showed risk prevalence 25% greater than the eutrophic. This fact was neither observed for the male population, nor in whole sample (Table 4). From Table 5 it comes that, among sociodemographic characteristics, gender and age group are related to FRS, an expected fact, since this factors are component variables of FRS.

**Table 4:** Prevalence ratio of FRS high risk in relation to BMI

BMI	General	Gender				
		Male		Female		
	PR (CI 95%)	p	PR (CI 95%)	p	PR (CI 95%)	p
Recommended range	1	-	1	-	1	-
Overweight	1.035 (0.86-1.25)	0.719	0.961 (0.74-1.24)	0.762	1.147 (1.03-1.27)	0.010
Obesity	1.052 (0.87-1.28)	0.610	1.067 (0.79-1.44)	0.672	1.25 (1.13-1.38)	< 0.001

FRS: Framingham Risk Score; BMI: body mass index; PR: prevalence ratio; CI: confidence interval.

**Table 5:** Relation between sociodemographic characteristics and the Framingham Risk Score

		FRS			p*
		Low risk** 97 (70.3)	Intermediate risk** 13 (9.4)	High risk** 28 (20.3)	
Gender					
	Male	27 (27.7)	12 (92.3)	13 (46.4)	< 0.001
	Female	70 (72.2)	1 (7.7)	15 (53.6)	
Age range					
	< 40 years	20 (20.6)	-	5 (17.9)	
	40-55 years	53 (54.6)	3 (23.1)	5 (17.9)	< 0.001
	> 55 years	24 (24.7)	10 (76.9)	18 (64.3)	
Marital status					
	Single	10 (10.3)	-	4 (14.3)	
	Married	74 (76.3)	12 (92.3)	18 (64.3)	0.382
	Others	13 (13.4)	1 (7.7)	6 (21.4)	
Economic level					
	B1-B2	43 (44.3)	4 (30.8)	11 (39.3)	0.615
	C1-C2-D	54 (55.7)	9 (69.2)	17 (60.7)	
Schooling					
	Up to 7 years	69 (71.1)	11 (84.6)	21 (75.0)	
	8 to 10 years	12 (12.4)	1 (7.7)	2 (7.1)	0.806
	11 years or more	16 (16.5)	1 (7.7)	5 (17.9)	

* Pearson's chi-square;

** frequency (percentage); FRS: Framingham Risk Score.

## DISCUSSION

The results of this cross-sectional study show the prevalence of overweight and obesity among workers classified with intermediate and high cardiovascular risk. Regardless of overweight or obesity present in most of sample, the subjects were often classified with low cardiovascular risk. From the factors considered in the FHS, the ones who obtained the highest scores in the formation of the risk score were age and TC. Variables that may be related because the increase in TC may be associated with increased age, as in this study the subjects had higher mean age (51.32 years) compared with those obtained by workers in other economic sectors, such as studies industrialists (36.27 ± 10.21) (17) and commercial workers (27.65 ± 9.38) (18).

This mean age is characteristic of the demographic profile of Brazilian rural workers marked by continuous aging (19). This phenomenon stems from the migration of youth to urban centers, as has also been identified in France (20). This happens due to the migratory flow, which is common in contemporary societies, according to Dasre and cols. (21).

However, in addition to issues related to the logic of the organization and dynamics of production centered on family labor, factors that contribute to male dominance is the aging of the rural population. However, external elements to the family play an important role in the movement of young people to the cities, such as the increasing difficulty of access to basic health and education services (22).

With respect to the average value of TC, it was observed that this was above the recommended as desirable in half the subjects in this study. This result shows resemblance to a survey of urban bus drivers in Teresina (Piauí), in which approximately half of subjects had TC at levels above 200 mg/dL (10), as well as 39.4% of 678 drivers, working in alternating shifts in a mining company in Minas Gerais, had increased TC (23). A bi-racial study with women and African-American and white men, found above the recommended cholesterol (> 200 mg/dL) among white women (24).

Regarding HDL-c most of the subjects were classified at levels considered adequate (50.03 ± 10.96). Similar average results were found in a study applied with 2037 subjects (24). It is important to highlight that the small number of smokers (6.5%) led to the disregard of this variable in this study.

Although the highest percentage of subjects has presented systolic blood pressure in the proper range, a third of them (33.1%) was classified with hypertension. This finding may be associated with the age of the workers, since study in two rural communities identified 42.9% of hypertensive in 18-94-year-old subjects (25). These findings confirm the scientific evidence that high blood pressure increases progressively with age, being a common condition in people with older age, especially in people over 60 years old (26). The Framingham Heart Study states that the prevalence of hypertension increases from 27.3%, in patients younger than 60 years, to 74.0% in those aged over 80 years old (27).

In addition to the primary prevention, it is necessary more control and treatment of hypertension, especially in critically ill patients, as shown in a study of medical records of patients from the Cardiology Clinic of Anapolis/GO. The same authors stress the urgency of greater control over risk factors, such as physical inactivity and obesity, in order to avoid the emergence of CVD associated with SAH (28).

This recommendation is based on the influence of overweight in the prevalence of hypertension due to the risk that these changes represent a condition found in 87.8% of workers. This finding overcomes the data from a study that observed overweight and obesity in 65.5% of subjects in the hospital area (29) and 40.7% of farmers in Minnesota, United States (30). It is possible to observe that one of the factors prevalent among hypertensive individuals is the obesity (31).

Regarding BMI, studies show that the prevalence of most chronic diseases are associated with the increase of this anthropometrical measure. In addition, the cardiometabolic risk factors have been affecting both men and women with overweight and obesity (32), with this measure related to increased risk of premature death in subjects with severe obesity or above 35 kg/m^2^ (33).

It is worth noting the risk posed by visceral fat and body fat for the development of coronary atherosclerosis, before the presence of comorbidities resulting from cardiovascular diseases (34). Thus, the Framingham score, for its ability to anticipate the identification of individuals at risk of developing cardiovascular events, is an important method for the primary prevention. Preventive interventions should be proposed and are indispensable in order to prevent the occurrence of any event.

Regarding the groups, no significant differences were observed between the clinical variables. According to the Framingham risk score, the average value found was 10.93 points, featuring intermediate risk of coronary events in 10 years. The results found in this study show that 22.3% of the subjects are at high risk of developing coronary event. Unlike the study of 309,955 workers in different economic sectors in Spain, with an average age of 36.5 years, in which 5.9% of the subjects were classified with high risk and 0.9% with moderate risk, those classified with high cardiovascular risk 7.6% were men and 1.7% were women, with a higher occurrence in agricultural (11.3%) and construction workers (8.2%), when compared to the industrial and services sector (35).

Finally, it is worth noting the presence of abnormal glucose (59.0%) with diabetic (15.8%) and pre-diabetic patients (43.2%) in the group assessed, highlighting the importance of primary actions that may reverse health complications through the assessment of cardiovascular risk in rural workers, using the Framingham risk Score, and stratify its distribution according to the classification of body mass index.

We considered the high number of diabetics in the sample was due to several factors, such as the advanced age of the individuals, the great number of overweight subjects and the fact that they referred not having regular attendance for this marker. Another point to be highlighted is the fact that men does not present risk increase as BMI increase. This fact points to weakness of BMI for part of this group, which could have been influenced by factors as lean mass increasing, what is not discriminated by BMI. Thus, we are sendend towards further investigations related to body composition and cardiovascular risk addressing the specifities of this population, especially regarding to the high average age.

The subjects of the study have a labor fairly active, mainly the men, which are more involved in heavy farm activities. Thus, men BMI might be biased, which could be explained by the overweight caused by lean body mass instead of fat body mass, since BMI does not tell which kind of body mass. This hypothesis could be investigated in future researches, using different anthropometric variables.

This study showed the presence of low risk for coronary events in the study population, estimated by the Framingham Risk Score. The influence of BMI on increasing cardiovascular risk was observed in the women workers. However, this fact was not identified in men. Neverthless, these results show the importance of this variable when implementing actions that can stimulate healthier lifestyles, especially when addressed to a population with limited access to health care networks, as in the case of rural workers.
